# Selectivity of Tungsten Oxide Synthesized by Sol-Gel Method Towards Some Volatile Organic Compounds and Gaseous Materials in a Broad Range of Temperatures

**DOI:** 10.3390/ma13030523

**Published:** 2020-01-22

**Authors:** Simonas Ramanavičius, Milda Petrulevičienė, Jurga Juodkazytė, Asta Grigucevičienė, Arūnas Ramanavičius

**Affiliations:** 1 Center for Physical Sciences and Technology, Sauletekio av. 3, LT-10257 Vilnius, Lithuania; simonas.ramanavicius@ftmc.lt (S.R.); milda.petruleviciene@ftmc.lt (M.P.); jurga.juodkazyte@ftmc.lt (J.J.); asta.griguceviciene@ftmc.lt (A.G.); 2 Department of Physical Chemistry, Faculty of Chemistry and Geosciences, Vilnius University, Institute of Chemistry, Naugarduko 24, LT-03225 Vilnius, Lithuania

**Keywords:** non-stoichiometric tungsten (VI) oxide (WO_3−x_), gas sensors, sol-gel technique, volatile organic compounds (VOC), reducing gases, temperature dependent sensitivity and selectivity

## Abstract

In this research, the investigation of sensing properties of non-stoichiometric WO_3_ (WO_3−x_) film towards some volatile organic compounds (VOC) (namely: Methanol, ethanol, isopropanol, acetone) and ammonia gas are reported. Sensors were tested at several temperatures within the interval ranging from a relatively low temperature of 60 up to 270 °C. Significant variation of selectivity, which depended on the operational temperature of sensor, was observed. Here, the reported WO_3_/WO_3–x_-based sensing material opens an avenue for the design of sensors with temperature-dependent sensitivity, which can be applied in the design of new gas- and/or VOC-sensing systems that are dedicated for the determination of particular gas- and/or VOC-based analyte concentration in the mixture of different gases and/or VOCs, using multivariate analysis of variance (MANOVA).

## 1. Introduction

Air pollution is an important environmental issue due to intensive industrial manufacturing processes, car exhaust, biomass burning, combustion of fossil fuels, and microbiological emission from soil. The main air pollutants such as NO_x_, SH_2_, NH_3_, and some volatile organic compounds (VOC) contribute to a greenhouse effect and can cause respiratory diseases such as bronchitis, emphysema, as well as heart problems [1]. Therefore, the demand for new sensors, which are suitable for the determination of gases and/or VOCs, is constantly increasing. In the development of gas- and/or VOC-sensors, metal oxides such as TiO_2_, SnO_2_, WO_3_, and ZnO are widely used as sensing components of analytical signal transduction systems, because of their high sensitivity and stability [2,3]. Among many other oxides, tungsten (VI) oxide (WO_3_) is a very attractive material due to its relative chemical stability, robustness, inherent electrical conductivity, and good sensitivity towards various gaseous materials including many VOCs [4,5,6]. Various WO_3_-based structures are also used in lithium-ion batteries [7] and solar energy conversion systems [8,9,10,11,12]. The gas- and/or VOC-sensing and photoelectrochemical properties of WO_3_ are highly dependent on the deposition method and conditions of sensitive layer formation. Different methods, such as reactive radiofrequency (RF) sputtering [13], hydrothermal synthesis [13,14], spray pyrolysis [15], electrodeposition [16,17], and sol-gel based surface modifications [18,19] are applied for the formation of WO_3_ films. Sol-gel technique for the formation of WO_3_ layers is among the most attractive ones, because this technique does not require any sophisticated and expensive equipment, the control of surface modification process is easy, and different variations of sol-gel based techniques can be applied for gas-sensing layer deposition on various substrates [19,20,21].

In the case of n-type semiconductors gas- and/or VOC-sensing occurs during the adsorption-desorption of gas- and/or VOC-molecules and/or semiconductor catalyzed reaction between the surface of sensor and gas- and/or VOC-molecules, which leads to the variation of their conductivity. Various WO_3_-based structures exhibit excellent sensing properties towards various oxidizing and reducing gases and/or VOCs and, therefore, different WO_3_-based structures can be used for particular safety-related applications, for example, the detection of indoor air pollutants, monitoring the toxic, flammable or explosive gases, and/or VOC in different environments. Some other sensing applications are related to medical diagnostics, e.g., the evaluation of exhaled acetone [22,23], etc. It is known that the performance of gas sensors is highly dependent on the morphology, crystallite dimensions, and porosity of WO_3_-based sensing layers. From this point of view, nanostructured materials are very attractive due to their large porous surface, which is important for the sensitivity of sensing layer.

The gas- and/or VOC-sensing mechanism is a complex process. It is based on the variation of WO_3_ layer resistance, which is affected by the gaseous materials, that are being detected, and the surface-concentration of the oxygen initially chemisorbed on the surface. In the process of O_2_ chemisorption, the electrons from the conduction band of WO_3_ are transferred to adsorbed O_2_ molecules, leading to the formation of ionic oxygen species [24,25]. Depending on temperature, different types of ionic oxygen species such as O_2_^−^, O^−^, O^2−^ can be formed [26]:

O_2_ (gas) → O_2_ (adsorbed on WO_3_) (1)

O_2_ (adsorbed on WO_3_) + e^−^ → O_2_^−^ at lower temperatures (< 150 °C) (2)

O_2_^−^ + e^−^ → 2O^−^ at higher temperatures (150–400 °C) (3)

O^−^ + e^−^ → O^2−^ at higher temperatures (> 400 °C) (4)

The electron transfer (ET) from conduction band of semiconductor to the chemisorbed oxygen results in the decrease of WO_3_-based layer conductivity, therefore, the registered resistance of WO_3_-based layer increases. Sensors based on tungsten oxide usually operate at higher temperatures, because the chemisorption of oxygen takes place at the temperature over 100 °C. Moreover, the temperature of sensing layer significantly influences the response of the sensor and even the selectivity of sensor towards different gases.

Depending on the type of gases (reducing or oxidizing), which are being determined by WO_3_-based sensors, electrons from the depletion layer are either transferred into the tungsten oxide based layer or are accepted by gas- and/or VOC-molecules, which are adsorbed on the surface of semiconducting layer.

The detection of reducing VOCs such as acetone occurs according to the following scheme [27]:

CH_3_COCH_3_ (gas) + O^−^ → CH_3_C^+^O + CH_3_O^−^ + e^−^ (5)

CH_3_C^+^O → C^+^H_3_ + CO (6)

CO + O^−^ → CO_2_ + e^−^ (7)

After the oxidation of adsorbed acetone molecules, electrons accepted by the tungsten oxide layer increase the electron concentration in the tungsten oxide based layer and, what consequently, turns into the decrease of WO_3_-based layer resistance.

Ethanol detection occurs according to the same mechanism (Equation (7)), i.e., transfer of electrons to the tungsten oxide based layer [28], such that the charge transfer process is leading to the decrease of the layer’s resistance:

C_2_H_5_OH (g) + O^−^ → CO_2_ (g) + H_2_O (g) + e^−^ (8)

Ammonia detection mechanism [29] follows the reactions provided below:

4NH_3_ + 5O_2_^−^ → 4NO + 6H_2_O + 5e^−^ (9)

2NH_3_ + 3O^−^ → N_2_ + 3H_2_O + 3e^−^ (10)

2NH_3_ + 3O^2−^ → N_2_ + 3H_2_O + 6e^−^ (11)

The detection of oxidizing gases such as CO_2_ or NO_2_ proceeds through opposite charge transfer direction [26] compared to the presented above for reducing gases (Equations (1–9)).

The goal of this study was to synthesize tungsten oxide based films with gas/VOC sensing properties and to evaluate the influence of temperature on response and selectivity of the sensor towards several VOCs and ammonia gas. Coatings based on a mixture of stoichiometric WO_3_ and non-stoichiometric WO_3−x_ (further in the text is denoted as WO_3_/WO_3−x_) were deposited on glass substrate using a simple and easily controllable sol-gel technique. Sensing properties of tungsten oxide films towards methanol, ethanol, acetone, and ammonia were evaluated in the broad range of temperatures. The kinetics of sensor response towards a flux of gaseous materials within the first 1 s was assessed and interpreted as an analytical signal. Analytical signals of WO_3_/WO_3−x_ -based sensor at different temperatures were compared.

## 2. Experimental

### 2.1. Chemicals

Sodium tungstate dihydrate (Na_2_WO_4_ × 2H_2_O) were purchased from Carl Roth (Karlsruhe, Germany), ammonium oxalate ((NH_4_)C_2_O_4_) (AO), HCl, and hydrogen peroxide (H_2_O_2_) were received from Chempur (Piekary Slaskie, Poland), ethanol (EtOH) was purchased from Reachem (Bratislava, Slovakia). All chemicals were used as received from suppliers, without any further purification.

### 2.2. The Formation of Tungsten Oxide Based Gas- and VOC-Sensitive Layers on a Glass Substrate

WO_3_/WO_3−x_-based thin films were deposited on glass substrate by the sol-gel method in aqueous solution. Firstly, 1 × 2 cm glass slides were prepared and these slides were cleaned in ultrasonic bath with acetone, ethanol, and finally with deionized water for 15 min in each solution. During the sol-gel formation process Na_2_WO_4_ and (NH_4_)C_2_O_4_ (AO) were dissolved in distilled water and HCl was gradually added within 10 min under continuous stirring at 40 °C. Afterwards, within the next 10 min, H_2_O_2_ was gradually added into the above mentioned solution while maintaining a constant temperature in order to form peroxotungsten acid (PTA). In the next stage, EtOH was added as a reducing agent into the prepared PTA mixture (PTA + EtOH), which was used for the further formation of WO_3_/WO_3−x_-based coating. After 10 min cleaned glass slides were dipped into the above mentioned PTA+EtOH solution at ‘face-down position’ and incubated for 140 min. This WO_3_/WO_3−x_-based coating formation step was carried out in a water bath at a constant 85 °C temperature. After the formation of WO_3_/WO_3−x_-based coating on glass slides, the slides were gently rinsed by distilled water for 1 min and then dried in the drying oven at 40 °C for 10 h. Finally, WO_3_/WO_3−x_-modified slides were annealed in ambient atmosphere at 500 °C for 2 h. Gradually increasing heating (1 °C min^−1^) was applied, while initial heating temperature was 20 °C. Before coating procedure glass slides were sonicated in acetone, isopropanol and water for 15 min in each.

### 2.3. Deposition of Platinum Contacts Over Gas- and VOC-Sensitive Tungsten Oxide Layer

**Figure 1:**
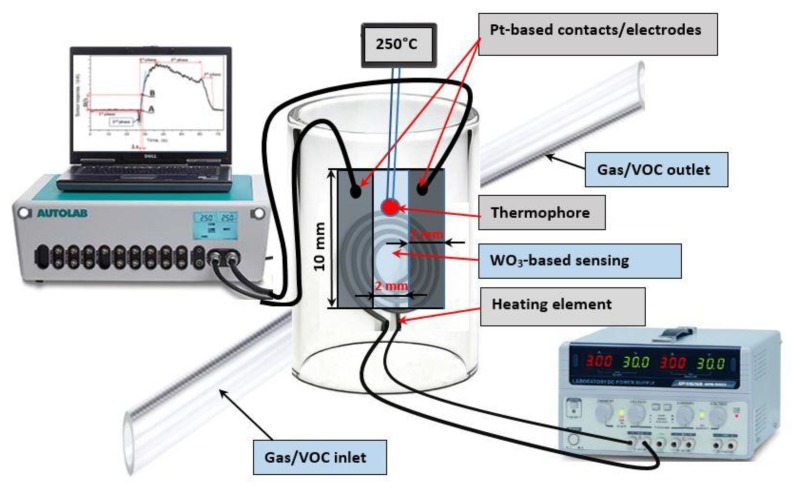
Major parts of experimental setup and structure of WO_3_/WO_3−x_-based sensor.

The magnetron sputtering system TFSP-840 from VSTSER (Tel-Aviv, Israel) was used for the sputtering of platinum contacts. Magnetron sputter operated in the DC regime under argon (Ar) atmosphere at 20 mTorr pressure. The geometry of contacts/electrodes was defined by a specially designed mask. In order to improve the adhesion between WO_3_/WO_3−x_-based coating and platinum, firstly a thin (20 nm) titanium (Ti) layer was sputtered on which Pt contacts were formed. Sputtering power for Ti layer formation was 2.55 Wcm^−2^ and titanium layer was formed at a rate of 0.13 nm·s^−1^. Platinum-based contacts/electrodes were formed over the Ti/WO_3_/WO_3−x_-based structure. The platinum target of 99.99% purity and of 2.5 cm diameter was used as a source of platinum. The dimensions of sesnor structure were as follows: 10 mm in length and 2 mm space between platinum contacts/electrodes, which were 2 mm wide (Figure 1). The magnetron power applied for the formation of platinum layer was 3.06 W·cm^−2^, which has provided a 0.08 nm·s^−1^ platinum layer formation rate. Sensor design and major parts of the experimental setup are presented in Figure 1.

### 2.4. Characterization of Oxide Layers

#### 2.4.1. Characterization by X-Ray Diffraction, Scanning Electron Microscopy

The X-ray diffractometer Smart Lab from Rigaku (Oxford, United Kingdom), which was equipped with a 9 kW rotating Cu anode X-ray tube, was used for the identification of crystalline phases in the sample. The grazing incidence (GIXRD) method was applied in a 2θ range 20–80 °C. An angle between a specimen surface (ω angle) and a parallel beam of X-rays was kept at 0.5 °C. Package PDXL Rigaku (Tokyo, Japan) software and Crystallography Open Database (COD) were used for the identification of crystalline phases.

Helios NanoLab dual beam workstation with X-Max 20 mm^2^ energy dispersion spectrometer from Oxford Instruments (Oxford, United Kingdom) was applied for the investigation of tungsten oxide coating morphology.

#### 2.4.2. Gas and VOCs Sensing Measurements

The sensor chip was attached from the back side of the chip (Figure 1). WO_3_/WO_3−x_-based gas sensors were tested at several different temperatures (namely at 60, 120, 180, 210, 250, and 270 °C), in the presence of the same concentrations of VOCs (methanol, ethanol, isopropanol, acetone) and ammonia gas. Gas flow rate measured in standard cubic centimetres per minute (SCCM) was 100 cm^3^/min.

The generation of VOCs and NH_3_ containing air stream was performed by a homemade system, consisting of VOCs containing/generating systems, pipes, valves, and gas chamber in which sensors were deposited and locally heated up. Heating was applied by an external heater, which was attached to the sensor chip from the bottom, while the temperature of sensing area was measured by a thermophore (Figure 1), which was attached to the side of sensor chip and connected to the display. Temperature control was performed by a manual adjustment of current passing through the heating element and following indications, which were provided by a thermophore connected to the display.

All experiments were performed at a stable concentration of VOCs, which were in the range of 10 ppm for all here evaluated VOCs (methanol, ethanol, isopropanol, acetone) and ammonia gas.

Potentiostat/galvanostat Autolab 30 from Ecochemie (Utrecht, The Netherlands) in two-electrode modes was used in all described amperometric measurements for the registration of WO_3_/WO_3−x_-based sensor response. The measurement of current passing through the sensing system (Figure 1) was performed at 750 mV constant voltage applied between electrodes. Electrical resistance decreased due to increased conductivity of WO_3_/WO_3−x_-based VOC-sensitive layer, therefore, the current passing through WO_3_/WO_3−x_-based layer increased.

In this research analytical signals were evaluated in two different ways:

*(1) Assessment of steady-state based analytical signal*

**Figure 2:**
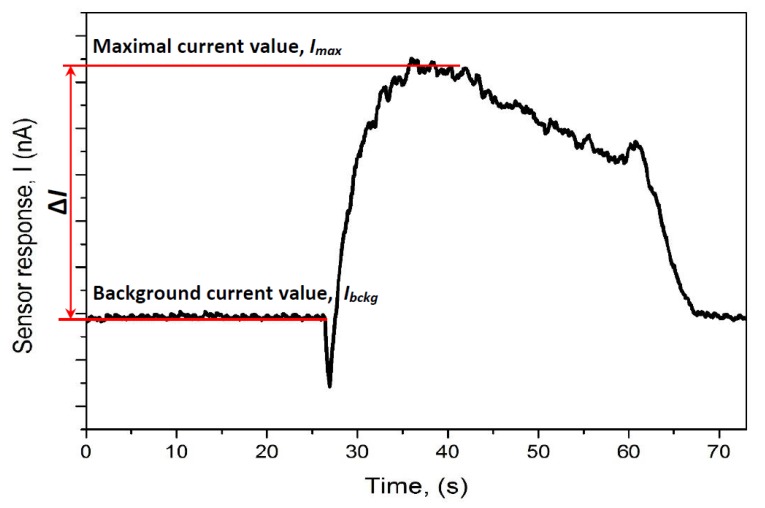
Assessment of steady-state based analytical signal (Δ*I*) from a typical WO_3_/WO_3−x_-based sensor response towards vapor of different VOCs (methanol, ethanol, isopropanol, acetone) and ammonia gas at temperatures between 60 and 270 °C.

Assessment of steady-state response (Δ*I*_X*,* T_) towards particular gas or VOC (here indexed as ‘X’) at temperature (here indexed as ‘T’) of the WO_3_/WO_3−x_-based sensor has been determined as the difference of current (Δ*I*) between a background current (*I*_bckg_) and current registered when signal reached its maximal value (*I_max_*) (Figure 2). Then, in order to compare all signals towards different VOC/gases at different temperatures the normalization was performed versus the highest signal, which was registered for methanol at 250 °C (Δ*I*_Methanol, 250 °C_), and the normalized signal (*R*_X, T_) was calculated for each particular gas and VOC (here indexed as ‘X’) at particular temperature (here indexed as ‘T’), *R*_X_, _T_ (in %) values was calculated in the following way:

*R*_X, T_ = 100 × Δ*I*_X, T_/Δ*I*_Methanol, 250 °C_ (%) (12)

*(2) Assessment of analytical signal based on response kinetics*

**Figure 3:**
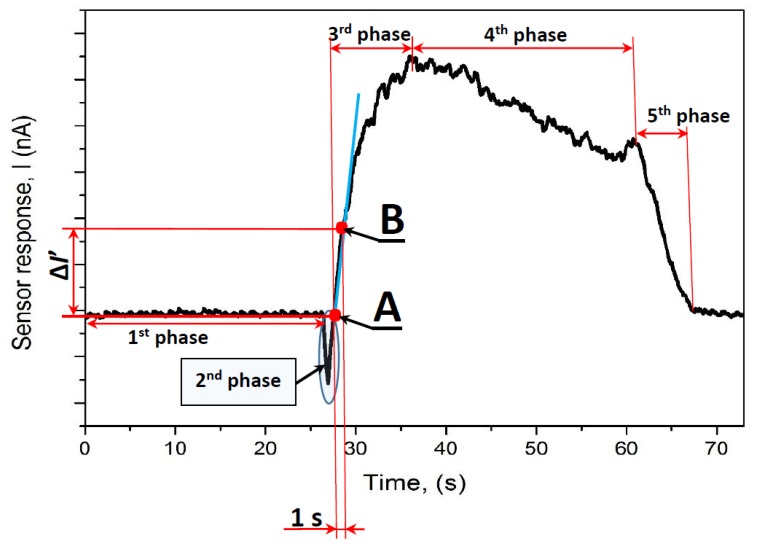
Assessment of kinetics-based analytical signal (Δ*I′*) from typical WO_3_/WO_3−x_-based sensor response towards vapor of different VOCs (methanol, ethanol, isopropanol, acetone) and ammonia gas at temperatures between 60 and 270 °C along with a scheme of Δ*I′* calculation, which indicates the increase of current during 1 s lasting period.

During the assessment of kinetics-based analytical signal (Δ*I′*), sensor response was divided into several steps:

(i) Background current (baseline) was achieved in the air (Figure 3, first phase);
(ii) Then, gas was quickly exchanged in the cell and short term 1–5 s (dependent on gas or VOC and applied temperature), amperometric signal decrease was observed, this short-lasting second phase, which was induced by a very fast exchange of analyte-free air by air-based aliquot containing analyte (gas or VOC), this part was not accounted into the analytical signal;
(iii) Current increase rate (Δ*I′*_X, T_) towards particular gas or VOC (here indexed as ‘X’) at particular temperature (here indexed as ‘T’); Δ*I′*_X, T_ was calculated as a change of the current within one second of measurement between points A and B, which are indicated in Figure 3; the calculation of current increase (Δ*I′*_X, T_) between point A (Figure 3, point A), which corresponds to the point when amperometric signal is passing the baseline value after the short-lasting second phase and point B (Figure 3, point B), which corresponds to the point registered during 1 s of the registration of point A.

In order to compare all signals to different gases at different temperatures the normalization was performed versus the highest signal, which was registered for methanol at 250 °C (Δ*I’*_Methanol, 250 °C_), and the normalized signal (*K*_X, T_) was calculated for each particular gas and VOC (here indexed as ‘X’) at particular temperature (here indexed as ‘T’), *K*_X_,_ T_ (in %) values was calculated in the following way:

*K*_X, T _= 100 × Δ*I’*_X, T_/Δ*I*_Methanol, 250 °C_ (%) (13)

## 3. Results and Discussion

**Figure 4:**
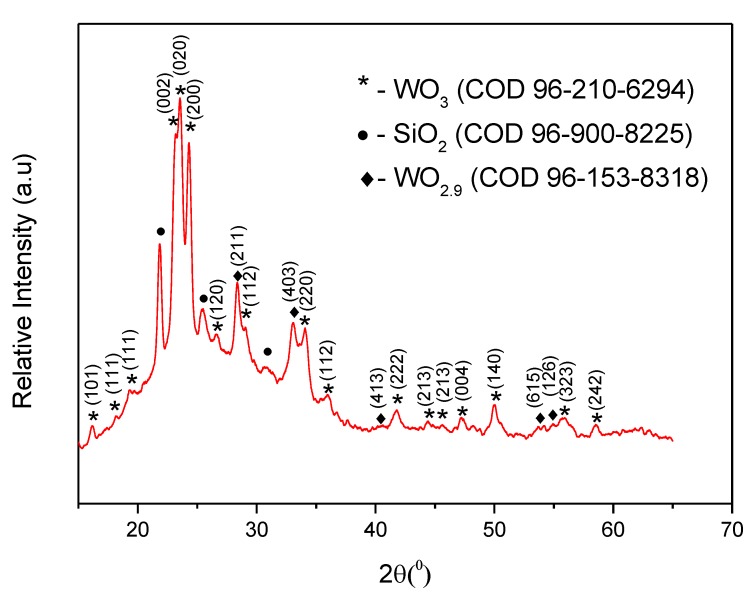
XRD spectrum of WO_3_/WO_3 − x_-based layer prepared from PTA + EtOH sol-gel and annealed at 500 °C for 2 h.

The crystalline structure and surface morphology of tungsten trioxide based coatings were characterized by X-ray diffraction (XRD) and scanning electron microscopy (SEM). Figure 4 shows XRD patterns of the sample prepared from PTA + EtOH sol-gel and annealed at 500 °C for 2 h. One can see, that the non-stoichiometric WO_3_/WO_3−x_ crystalline phase in synthesized coating is formed. The cluster of three characteristic diffraction peaks at 2θ = 23.15, 23.59, and 24.29° corresponds to monoclinic tungsten trioxide (Figure 4, peaks marked by asterisk) in accordance with PDF No. 96-210-6383 of the COD. Two clusters of two peaks at 2θ = 28.34, 29.04, 33.08, and 34.09° are attributed to monoclinic tungsten trioxide and non-stoichiometric monoclinic WO_2.9_ (Figure 4, peaks marked by ‘diamond’). Since WO_3_/WO_3−x_-based layers are very thin, some XRD-features of substrate are also present in XRD spectra. Three peaks at 2θ = 21.83, 25.49, and 30.74° (Figure 4, peaks marked by circles) are attributed to the SiO_2_ substrate in accordance with PDF No. 96-900-8225 of the COD. The crystalline structure of tungsten oxide layer depends on annealing temperature. In some researches, it has been demonstrated that 500 °C is the most optimal temperature for the formation of crystalline monoclinic tungsten oxide based structures [30,31,32,33,34]. Phase transformation of tungsten oxide occurs in the following sequence: Monoclinic II (ε-WO_3_, below −43 °C) → triclinic (δ-WO_3_, between −43 to 17 °C) → monoclinic I (γ-WO_3_, 17 to 330 °C) → orthorhombic (β-WO_3_, 330 to 740 °C) → tetragonal (α-WO_3_ over 740 °C) [35]. Thermal gravimetric analysis (TGA) revealed two mass-loss steps: The first mass loss occurred below 100 °C and corresponded to the evaporation of absorbed water, it confirms the sensitivity of this material to water adsorption. The second mass loss occurs between 170 and 300 °C and is based on the removal of inter-structural water molecules [36,37,38,39].

**Figure 5:**
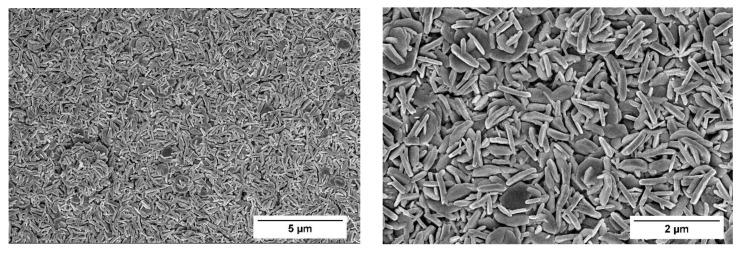
SEM images of WO_3_/WO_3 − x_-based sample prepared from PTA + EtOH sol-gel and annealed at 500 °C for 2 h. Left side: Magnification ×10,000; Right side: Magnification ×25,000.

Morphology of tungsten trioxide coatings was analyzed using the SEM technique. In Figure 5, SEM images of WO_3_/WO_3−x_-based layer at different magnification are presented. Coating is dense, composed of randomly oriented sub-micrometer-sized (200–1000 nm), nanorod-, nanowire-, and plate-shaped particles, which are located very close to each other forming the network with high surface area. SEM images reveal that tungsten trioxide coatings are evenly deposited without cracks in the structure, which enables efficient charge transfer during the gas- and/or VOC-sensing process. Some authors have reported that nanorod- or nanowire-shaped structures exhibit significantly higher sensitivity and stability for gas sensing with fast response-recovery time. In addition, they report, that the use of one-dimensional structures for gas sensor applications enhances sensor performance through the improvement of the active sensing sites [40]. These nanostructures provide suitable blocks for the development of high performance gas- and/or VOC-sensors [41]. Sensing layers based on perpendicularly oriented crystallites showed higher photoelectrochemical performance than that based on layers consisting of nanocrystalline particles [42].

Typical analytical signal towards analyte (in this case towards ethanol) is presented in Figure 2 and Figure 3. The increase of electrical conductivity is registered when the flux of ethanol vapour is delivered into the reaction chamber. Such response towards ethanol reveals that the WO_3_/WO_3−x_-based film is compact enough to provide sufficient conductivity. Different from many other researches [25,26,27,28,29,43,44,45], in our sensing element contacts/electrodes were deposited on the top of WO_3_/WO_3−x_-based film which provided better contact between platinum-based electrodes and upper layer of WO_3_/WO_3−x_, which probably is the most important for the sensing ability of such sensing structure. It should be also noted that the regeneration of sensor, which is determined by the recovery of the signal to the initial ‘baseline’, is relatively quick and the baseline is well recovered by replacement of analyte containing air sample after the measurement by clean air. The curve in Figure 3 can be divided into several district phases of measurement cycle listed further. The first phase represents the baseline, which is achieved before the measurement. The second phase represents a very short decrease of current during the flux of the VOCs through the inlet into the gas chamber. This signal flux is induced by a short-term increase in resistance of WO_3_/WO_3−x_-based layer, when oxygen, which was initially adsorbed on WO_3_ surface, is replaced by VOC-based analyte present in the aliquot, and initial rearrangements within WO_3_ grains are taking place. The third phase represents the increase in current measured amperometrically at a constant voltage of 750 mV. The increase in current is due to the oxidation of reducing gases and/or VOCs by catalytic action of WO_3_, which is followed by electron transfer into oxide layer. This increases the superficial conductivity of WO_3_ grains and, subsequently, leads to the increase in the conductivity of all sensing WO_3_/WO_3−x_-based layer. The fourth phase represents a slow decrease of current due to the passivation/inactivation of WO_3_/WO_3−x_-based layer surface by reaction products. The fifth phase represents the regeneration of WO_3_/WO_3−x_-based layer by air, which is reflected by a fast decrease of measured current due to the removal of gas and/or VOC-based analyte and reaction products by oxygen, which is present in air used for the regeneration of sensing layer.

According to the point of view of the discussed above specific features of analytical signal, it is not reasonable to evaluate the pseudo steady-state current of WO_3_/WO_3−x_-based layer, which appears at the end of the third phase in Figure 3 due to the following reasons: (i) This pseudo steady-state phase is relatively short and not well expressed, because the signal starts to decrease and turns into the fourth phase, which is characterized by continuous decay of analytical signal; (ii) it takes a relatively long time (5 to 60 s depending on the gas- and/or VOC-based analyte and applied heating temperature) until the signal reaches the end of the third phase, which for a very short period of time is just characterized by steady-state current. Therefore, in this research we have evaluated the kinetics of the initial increase of current in the third phase between the indicated points A and B (Figure 3, points A and B), because the evaluation of kinetics was the most reliable analytical signal. We have eliminated the decrease of current during the flux of the VOC, but we have used values of current and time at the initial point (Figure 3, point A) of this flux for the calculation of signal kinetics. The next point, which was used for the evaluation of kinetics was taken after 0.5 s (Figure 3, point B). The procedure described herein enabled us to reduce the time, which is required for the registration of analytical signal, because it was not necessary to wait for the evolving of complete analytical signal and, more importantly, due to shorter exposure to VOC-based analyte, the tendency of WO_3_/WO_3−x_-based gas- and VOC-sensitive layer to degrade has also significantly decreased.

Different from the analytical signal registration methodology proposed by Yuan et al. [29] or by Horsfall et al. [28], where authors measured and evaluated the steady-state signal, for which they needed (i) at least 100 s for sensor response and (ii) additional 45 s for the regeneration of sensor and the recovery of ‘baseline’, our measurement together with recovery time lasted just several seconds. Additional advantage related to the assessment of initial kinetics compared to the evaluation of steady-state signal is based on specific features of chemical reactions, which occur during catalytic oxidation based sensing of some here evaluated VOC [46]. Different from the general scheme represented in the Introduction (Equation (7)), these reactions proceed through several distinct steps, which are presented below in Equations (12–17). At higher than room temperatures in the presence of air the majority of gas- or VOC-molecules, which are adsorbed on the metal oxide-based surfaces, are oxygen species (O^−^) [47,48]. It has been shown that electrical resistance of metal oxide structures at high concentrations of reducing gases and/or VOCs depends on the type of the oxygen-based species formed on WO_3_/WO_3−x_-based layer. Therefore, the sensing mechanism of ethanol is based on several steps [49]:

C_2_H_5_OH_(vap.)_ + O_(ads)_^−^ ↔ CH_3_CHO_(ads)_ + H_2_O_(vap)_ + e^−^ (14)

The electron released during oxidation of ethanol (Equation (12)) is injected into ‘the bulk’ of WO_3_/WO_3−x_-based layer. The injection of electrons generated during this process (Equation (12)) induces the increase of conductivity of WO_3_/WO_3−x_-based layer, which was registered as an increase of amperometrically registered current. Then, the product (acetaldehyde) of the above reaction is oxidized on the surface of WO_3_, which results in the formation of an oxygen vacancy (V_O_) [50]:

CH_3_CHO_(ads)_ + O_(bulk_↔ CH_3_COOH_(vap)_ + *V*_O_ here *V*_O_ is the oxygen vacancy (15)

Moreover, when the concentration of adsorbed oxygen significantly decreases, then, additional oxidation mechanisms are involved into WO_3_/WO_3−x_-catalysed oxidation of ethanol [46]:

C_2_H_5_OH_(vap)_ + W_(lat)_ → W_(lat)_OC_2_H_5_ + H_(ad)_^+^ (16)

When WO_3_/WO_3−x_-based surface becomes sufficiently deficient in oxygen, then two C_2_H_5_O_(ad)_ are starting to interact with each other to produce ethyl ether (C_2_H_5_)_2_O_(vap)_ according to the reaction as follows:

C_2_H_5_OW_(lat)_O_(lat)_W_(lat)_OC_2_H_5_ → O_(ads)_^−^W_(lat)_O_(lat)_W_(lat)_ + (C_2_H_5_)_2_O_(vap)_ + e^−^ (17)

Here, formed residual oxygen moiety (O_(ads)_^−^), which is included into the structure of O_(ads)_^−^W_(lat)_O_(lat)_W_(lat)_, reacts with the ethanol molecule and once O_(ads)_^−^ is consumed by ethanol oxidation reaction (Equation (12)), another cycle of ethanol oxidation reaction (Equation (15)) takes place with the next ethanol molecule, leading to the formation of new O_(ads)_^−^ species. The increase in electrical conductivity, which is observed as raising of amperometrically registered current, is also explained by the migration of some O_(ads)_^−^ species from the WO_3_ volume towards the surface of WO_3_/WO_3−x_-based layer, leading to the creation of additional oxygen vacancies in the bulk. Due to the reaction mechanism presented here (Equations (12–15)), the presence of oxygen in the surrounding atmosphere is required to determine a fast, well-reproducible, amperometric response of sufficient amplitude (Figure 3).

When the test chamber is purged by air and ethanol vapour is removed during the sensing-layer regeneration step, the accumulation of O_(ads)_^−^ species on the surface of WO_3_-based grains takes place, according to reaction equations represented below:

½ O_2(g)_ + e^−^ + *A*_s_ ↔ O_(ads)_^−^ here, *A*_s_ is the oxygen adsorption site on the WO_3_ surface (18)

O_(ads)_^−^ + *V*_O_^+^ ↔ O_(bulk)_ here, *V*_O_ is the oxygen vacancy (19)

Hence, both processes, i.e., adsorption of oxygen (Equation (16)) and the oxidation of WO_3_/WO_3−x_-based surface and bulk (Equation (17)) are taking place in a consecutive way during the regeneration of WO_3_/WO_3−x_-based layer by air. Therefore, the presence of oxygen in the surrounding atmosphere is critical for the regeneration of WO_3_/WO_3−x_-based layer after the determination of reducing organic VOCs. In such a way, adsorbed/integrated (Equations (16–17)) oxygen leads to the formation of electron depletion layer on the surface of WO_3_/WO_3−x_-based layer. When oxygen species are reacting with the reducing organic compounds, then the injection of electrons is narrowing ‘the width’ of the electron depletion layer, which is leading to the decrease in resistance of sensing layer. Corresponding chemical reactions between analyte-molecules and oxygen species are presented in Equations (1–15) [51,52].

The processes presented in Equations (13–15) are responsible for a constant drift of the baseline in the presence of ethanol, which is due to very slow kinetics are not reaching steady-state conditions for a very long time, exceeding 15 min according to some other authors [46]. Such long-lasting drift was also clearly observed in our research. The short steady state was observed only during the transition between third and fourth phases (Figure 3, third and fourth phases). Therefore, the evaluation of kinetics of the analytical signal, which from the point of view of chemical kinetics is the most dependent on the concentration of reacting gas and/or VOCs, is providing the most reliable analytical results.

**Figure 6:**
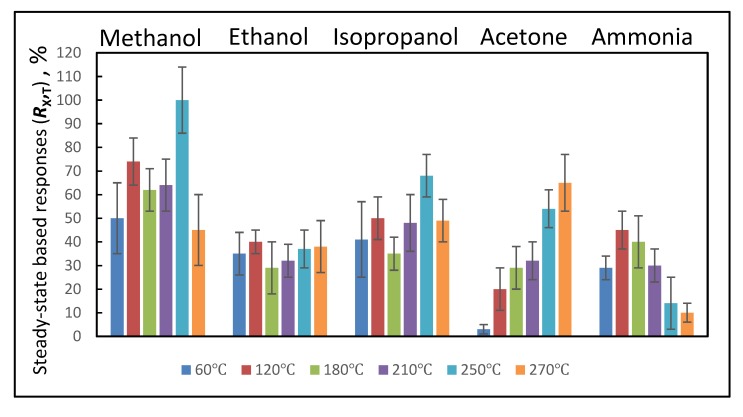
Normalized steady-state assessment based analytical signals (*R*_X_, _T_) of WO_3_/WO_3 − x_-based sensing layer towards VOCs (methanol, ethanol, isopropanol, acetone) and ammonia gas at 60, 120, 180, 210, 250, and 270 °C temperatures.

**Figure 7:**
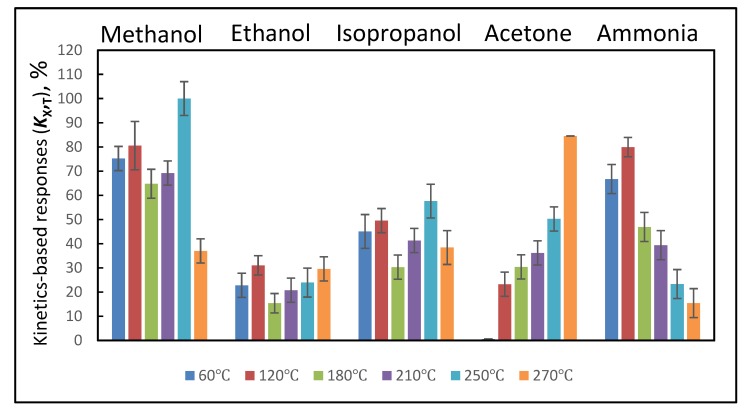
Normalized kinetics-based analytical signals (*K*_X_, _T_) of WO_3_/WO_3−x_-based sensing layer towards VOCs (methanol, ethanol, isopropanol, acetone) and ammonia gas at 60, 120, 180, 210, 250, and 270 °C temperatures.

Initial resistance of WO_3_/WO_3−x_-based layer was 3.75 G Ω at 270 °C. The response of WO_3_/WO_3−x_-based sensor towards fixed concentrations of evaluated VOCs (methanol, ethanol, isopropanol, acetone) and ammonia gas at 270 °C reveals relatively great sensitivity of WO_3_/WO_3−x_-based layer towards all VOCs tested (Figure 6 and Figure 7). In both figures we are representing normalized analytical signals, because the normalization of sensor signals is a typical approach, which is very often used for the representation of analytical signals during the assessment of sensor responses [53].

Some differences in normalized signal values are observed between data presented in Figure 6 and Figure 7, which provide differently assessed responses of WO_3_/WO_3−x_-based sensing layer towards VOCs (methanol, ethanol, isopropanol, acetone) and ammonia gas at temperatures between 60 and 270 °C. Despite some differences in normalized analytical signals presented in both figures (Figure 6 and Figure 7) the tendency remains the same. However, more precise comparison of presented data clearly shows that the assessment of steady-state based analytical signal—*R*_X, T_ (Figure 6), provides higher analytical signal discrepancies, which are indicated as standard deviations for each signal, in comparison to that observed during the assessment of analytical signal based on response kinetics—*S*_X, T_ (Figure 7). Moreover, the registration duration, which is required for the calculation of steady-state based analytical signal (*R*_X, T_), is several times longer in comparison to that, which is required for the calculation of response kinetics based analytical signal (*S*_X, T_), due to long-lasting achievement of steady-state conditions.

In the presence of VOC-based analyte, the analytical signal increased due to increased conductivity of sensing layer because all VOCs used in this research donated electrons to WO_3_ as described: (i) In Equations (4–6) for acetone, (ii) in Equations (7) and (12–15) for ethanol and the same can be adjusted for methanol and isopropanol, and (iii) in Equations (8–10) for ammonia. As can be seen from Figure 7, relatively simple dependences of analytical signal versus applied heating temperature is observed for acetone and ammonia, while for all three here evaluated alcohol-based VOCs (particularly methanol, ethanol, isopropanol) these dependences are more sophisticated due to very complex and temperature-dependent oxidation of alcohols on WO_3_/WO_3−x_-based sensing surface. It is interesting to note, that analytical response towards ammonia was the highest at 120 °C and it has systematically decreased with the increasing temperature of sensing surface. The tendencies for other VOCs were different. In the case of acetone at 60 °C the signal was just a few percent of that registered for methanol at 250 °C. It is noteworthy that the analytical signal has increased with the increasing temperature and it was the highest at a final tested temperature of 270 °C. Interestingly, the signal towards isopropanol was the highest at temperatures of 60 and 120 °C, while at 180 °C it dropped to some extent and then started to increase again with the rise of temperature and reached the maximum at 250 °C. A similar shape of temperature dependence with two maximums was observed for methanol, while for ethanol the lowest signal was observed at 180 °C, then it continuously increased with temperature and was the highest at the highest applied temperature of 270 °C.

In addition to all the above presented and discussed catalytic reactions between gas and/or VOC molecules and WO_3_/WO_3−x_-based layer surface (Equations (1–15)), the standard gas and/or VOC sensing process additionally involves the adsorption and desorption of gas- and/or VOC-based analyte and pre-adsorbed oxygen [54]. These processes (adsorption and desorption of VOC-based analyte and pre-adsorbed oxygen) are significantly affected by temperature. Therefore, as it is seen from our results (Figure 7) the sensitivity and especially the selectivity of WO_3_/WO_3−x_-based sensing surface is strongly dependent upon the applied operating temperature, which is in agreement with results presented by some other researchers [55]. Actually, the majority of reported WO_3_/WO_3−x_-based sensors are operating at relatively high temperatures in the range of 200–500 °C [2,22,28,56,57]. Such high temperatures are required to ensure the sufficient sensitivity. Our results clearly demonstrate (Figure 7) that the here reported WO_3_/WO_3−x_-based layer can be applied in the sensor, which operates at relatively low temperatures. It should be noted that the sensitivity towards VOCs and ammonia gas at low temperature of WO_3_/WO_3−x_-based layers has been recently observed only in several other researches, which are structurally very different because WO_3_-based sensing layers either were combined with conducting polymers (polypyrrole [6] and polyaniline [58]) or were prepared by completely different approaches [4,59]. These approaches enabled the formation of p-n hetero-junctions in WO_3_-based layers [4,6,58,59] or the non-stoichiometric p-type WO_3_ (WO_3−x_) was formed due to specific synthesis conditions. [4]. Remarkable properties of WO_3−x_-based layers, which are suitable for various technological applications, were reported in several researches, e.g., WO_2.72_ [60,61,62,63] and WO_2.625_ [4], which were applied in the design of low temperature sensors.

Therefore, the sensitivity of WO_3_/WO_3−x_-based layer, which is reported in the present research, towards here evaluated VOCs at relatively low temperatures (Figure 7) with temperature dependence containing several activity maximums in some cases implies that there is a switching between two very different WO_3_-based gas- and/or VOC-sensing mechanisms: (i) One based on n-type conductivity, which is characteristic for stoichiometric WO_3_ [28,53,64]; (ii) and the other based on p-type conductivity, which is a characteristic feature of non-stoichiometric WO_3_ (WO_3−x_). The latter was reported by Yao et al. [4] and can be responsible for the low-temperature sensitivity of such WO_3−x_-based layers towards gas and/or VOC-based analytes. In addition to the above discussed low-temperature sensitivity towards here evaluated VOCs, XRD results, which are presented in Figure 4, clearly illustrate the presence of some non-stoichiometric WO_3−x_, with stoichiometry of WO_2.9_, which provides p-type low-temperature sensitivity towards here evaluated VOCs due to increasing hole-based conductivity. The WO_2.9_ formed in our research shows the sensitivity towards reducing gases and/or VOCs at a slightly higher temperature (60 °C), compared to that reported by some other authors for WO_2.625_-based layer [4], which was also sensitive towards alcohols even at room temperature (25 °C). It is known that active sites available for the adsorption of VOC-based analyte molecules are based on oxygen vacancies. As it is presented in Equation (16), oxygen molecules from air can be adsorbed onto the WO_3_-based surface and form catalytically active oxygen species (Equation (17)). The type of dominating oxygen species, which is pre-determined by the operating temperature, is influencing significant variations in the resistance of WO_3_-based sensing layer.

It should be noted, that at lower temperatures there is a lower surface-concentration of ionized oxygen species on the WO_3_-based surface in comparison to the surface-concentration of these species formed at higher temperatures. Therefore, at lower temperatures, stoichiometric WO_3_-based sensing layer normally does not show obvious resistance change towards reducing gases and/or VOCs [65]. However, different from stoichiometric WO_3_-based surfaces the oxygen-deficient surface of non-stoichiometric WO_3−x_ is binding molecules of gas and/or VOC molecules more effectively, thereby, WO_3−x_-based layers are generating significant changes in electrical resistance, what was observed in our research for almost all evaluated reducing VOCs (Figure 7, at temperatures of 60 and 120 °C) only with one exception—for acetone the tendency of electrical resistance variations was different (Figure 7). While at higher temperatures in our research, the reported WO_3_/WO_3−x_-based layer switched to a ‘standard’ n-type conductivity-based sensing mechanism. Therefore, at intermediate temperature (180 °C), in the range between domination of p- and n-type conductivities, the decrease of analytical signal for methanol, ethanol, and isopropanol was observed. At higher temperatures the sensitivity towards mentioned reducing gases and/or VOCs increased again due to specific feature of n-type WO_3_-layer to provide higher sensitivity at temperatures that are close to 300 °C. When the temperature of WO_3_-based layer was raised above 200 °C, the WO_2.9_ gradually oxidized into WO_3_, which is characterized by a ‘standard’ n-type conductivity-based sensing mechanism. This observation is in line with the effect, which was observed for p-type urchin-like WO_3−x_ (WO_2.625_) nanostructures when the temperature of WO_3−x_-based sensing layer has increased over 200 °C [4]. P- and n-type conductivity of semiconductors is determined by dominating charge carriers (electrons or holes) in semiconducting material [66,67]. Therefore, observed variations of selectivity of here reported WO_3_-based sensing layer can be related to switching between p- and n-type conductivities, which are dominating at low and high temperatures, respectively. It should be noted that adsorbed water can play a significant role in the room-temperature based sensing of gases and/or VOC such as it was reported for some other metal oxide (e.g., ZnO) based layers [68,69,70,71,72].

## 4. Conclusions and Future Trends

Tungsten oxide films in the form of densely packed plate-shaped particles were synthesized by the sol-gel method and their selectivity towards methanol, ethanol, isopropanol, acetone, and ammonia was tested in a broad range of temperatures. Kinetics of the current increase at a constant 750 mV voltage was evaluated in order to obtain fast, well-reproducible, analytical signal with sufficient amplitude. The investigation of VOCs sensing properties revealed that the sensitivity and selectivity of WO_3_/WO_3−x_-based film varies significantly depending on the applied temperature of sensing structure. At 270 °C the WO_3_/WO_3−x_-based layer was not very sensitive to ammonia, while at 60 °C it was still very sensitive towards methanol. Relatively simple dependences of analytical signal versus applied heating temperature were observed for acetone and ammonia, while for the evaluated alcohol-based VOCs (methanol, ethanol, isopropanol) these dependences are more sophisticated due to very complex and temperature-dependent oxidation of alcohols on the WO_3_/WO_3−x_-based sensing surface.

The significant advantage of here reported WO_3_/WO_3−x_-based layer is that it does not require a high operating temperature, as it can decrease the stability of sensing layer, increase power consumption, and significantly raise safety concerns related to possible ignition of flammable and explosive gas- and/or VOC-based analytes.

Such WO_3_/WO_3−x_-based layers open an avenue for the design of sensors with temperature dependent sensitivity and selectivity that can be applied in the design of gas- and/or VOC-sensing systems, which are dedicated to the determination of particular gas and/or VOC concentration, using multivariate analysis of variance (MANOVA). Therefore, in the near future we are planning to design a sensing system consisting of here reported WO_3_/WO_3−x_-based layers operating at different temperatures and to apply MANOVA for the calculation of gas and/or VOC concentrations in the mixtures of gases and/or VOCs tested in this research.
